# Risk factors of multimorbidity among older adults in India: A systematic review and meta‐analysis

**DOI:** 10.1002/hsr2.1915

**Published:** 2024-02-28

**Authors:** Nikita Goel, Isha Biswas, Kaushik Chattopadhyay

**Affiliations:** ^1^ Lifespan and Population Health, School of Medicine University of Nottingham Nottingham UK; ^2^ The Nottingham Centre for Evidence‐Based Healthcare: A JBI Centre of Excellence Nottingham UK

**Keywords:** chronic diseases, elderly, health conditions‐related factors, India, lifestyle factors, meta‐analysis, multiple long‐term conditions, senior citizen, sociodemographic factors, systematic review

## Abstract

**Background:**

Multimorbidity among older adults is a growing concern in India. Multimorbidity is defined as the coexistence of two or more chronic health conditions in an individual. Primary studies have been conducted on risk factors of multimorbidity in India, but no systematic review has been conducted on this topic. This systematic review aimed to synthesize the existing evidence on risk factors of multimorbidity among older adults in India.

**Methods:**

The JBI and Preferred Reporting Items for Systematic Reviews and Meta‐Analysis guidelines were followed. Several databases were searched for published and unpublished studies until August 03, 2022. The screening of titles and abstracts and full texts, data extraction, and quality assessment were conducted by two independent reviewers. Any disagreements were resolved through discussion or by involving a third reviewer. Data synthesis was conducted using narrative synthesis and random effects meta‐analysis, where appropriate.

**Results:**

Out of 8781 records identified from the literature search, 16 and 15 studies were included in the systematic review and meta‐analysis, respectively. All included studies were cross‐sectional, and 10 met a critical appraisal score of more than 70%. Broadly, sociodemographic, lifestyle, and health conditions‐related factors were explored in these studies. The pooled odds of multimorbidity were higher in people aged ≥70 years compared to 60‐69 years (odds ratio (OR) 1.51; 95% confidence interval (CI) 1.20–1.91), females compared to males (1.38; 1.09–1.75), single, divorced, separated, and widowed compared to married (1.29; 1.11–1.49), economically dependent compared to economically independent (1.54; 1.21–1.97), and smokers compared to non‐smokers (1.33; 1.16–1.52) and were lower in working compared to not working (0.51; 0.36–0.72).

**Conclusion:**

This systematic review and meta‐analysis provided a comprehensive picture of the problem by synthesizing the existing evidence on risk factors of multimorbidity among older adults in India. These synthesized sociodemographic and lifestyle factors should be taken into consideration when developing health interventions for addressing multimorbidity among older adults in India.

## 1 INTRODUCTION

By the end of the century, one‐third of India's population will be aged 60 or more years,^1^ increasing the risk of multimorbidity.^2^ Multimorbidity is the coexistence of two or more chronic health conditions in an individual, each one of which is either a physical non‐communicable disease (NCD) of long duration (e.g., cardiovascular disease), a mental health condition of long duration (e.g., dementia), or an infectious disease of long duration (e.g., hepatitis C), and this is most widely used definition.^3^, ^4^ In India, the prevalence of multimorbidity among people aged ≥60 years ranges from 24% to 83%.^5^ Multimorbidity poses a significant burden on the healthcare system and the economy along with having a negative impact on the patients and their families and carers.^6^ People with multimorbidity require complex management regimens that may include polypharmacy, unplanned or emergency hospital visits, hospital admissions and re‐admissions, prolonged hospital stays, and out‐of‐pocket expenditures for drugs and healthcare services.^7^, ^8^, ^9^, ^10^ Major health consequences of multimorbidity include negative effects on the physical and mental health of patients such as physical disabilities, psychological distress, cognitive impairment, self‐doubts, depression, and anxiety.^7^, ^11^ Their capacity to perform activities of daily living may progressively deteriorate^12^, ^13^ hence, leading to poor quality of life and eventually leading to premature deaths.^14^, ^15^, ^16^, ^17^

In India, several primary studies have been carried out to identify risk factors of multimorbidity among older adults,^1^, ^18^, ^19^, ^20^, ^21^, ^22^, ^23^, ^24^, ^25^, ^26^, ^27^, ^28^ but to date, no systematic review has been conducted on this topic that brings together all the available evidence. The findings could be used when developing health interventions for addressing multimorbidity among older adults in India. Therefore, this systematic review aimed to synthesize the existing evidence on risk factors of multimorbidity among older adults in India.

## 2 METHODS

The systematic review was conducted and reported in conformity with JBI systematic reviews of etiology and risk and Preferred Reporting Items for Systematic Reviews and Meta‐Analysis (PRISMA) guidelines.^29^, ^30^ The review protocol was registered with PROSPERO (registration number: CRD42022348425).

### 2.1 Inclusion criteria

#### 2.1.1 Population

The systematic review included studies conducted among older adults (aged ≥60 years) in India. The phrases “senior citizen” or “elderly” are used in India to refer to those ≥60 years, as per the National Policy on Older Persons and Social Statistics Division of the National Statistics Office.^31^, ^32^ UN also defines the population aged ≥60 years as being old.^33^ If a study was conducted among adults, relevant data on older adults were extracted. The study was excluded if it was not possible to extract these data. Any study setting was eligible, for example, community, residential care, primary care, secondary care, and tertiary care.

#### 2.1.2 Exposure

Studies reporting any risk factor, such as sociodemographic (e.g., age and sex), lifestyle (e.g., smoking and alcohol consumption), and health conditions‐related, were included in this systematic review.

#### 2.1.3 Outcome

Studies on multimorbidity as an outcome were included in this systematic review. The study authors' definition of multimorbidity was used for this purpose. If the term multimorbidity or its definition was not mentioned, an operational concept of multimorbidity was used, that is, “coexistence of two or more chronic health conditions”.^3^ Studies on comorbidity, defined as “any distinct additional entity that has existed or may have occurred during the clinical course of a patient who has index disease under study”, were excluded.^3^, ^34^

#### 2.1.4 Study design

This systematic review included analytical observational studies (e.g., cohort, case‐control, and cross‐sectional studies).

### 2.2 Information sources and search strategies

Several databases were searched on August 03, 2022. The electronic databases searched for published articles were: MEDLINE (OVID; since 1946), EMBASE (OVID; since 1974), PsycINFO (OVID; since 1806), and CINAHL Plus (EBSCOhost; since 1945). For gray literature, ProQuest Dissertations and Theses was searched. The search strategies were developed based on previous similar systematic reviews^5^, ^35^, ^36^, ^37^, ^38^ and in consultation with a librarian (see Supplementary Materials). No date or language restrictions were applied. The reference list of all included studies and relevant systematic reviews were screened to identify any additional studies.

### 2.3 Study selection

Following the search, identified citations were collated and exported using a reference manager software, Endnote X9.^39^ Once duplicates were removed, records were imported into a web tool for systematic reviews, Rayyan.^40^ Titles and abstracts were screened against the eligibility criteria by two independent reviewers (NG and IB). Studies identified as potentially eligible or those without an abstract were retained for the full‐text screening. If the full text of an article was not available even through the interlibrary loan service at the University of Nottingham (UK), the corresponding author was contacted for the same (at least twice via email). For eligibility assessment, the full text of the articles was screened independently by two reviewers (NG and IB). Any disagreements that arose between them were resolved through discussion. If a consensus was not reached, a third reviewer (KC) was involved. Following the full‐text screening, studies not fulfilling the inclusion criteria were excluded, and reasons for the same were recorded (see Supplementary Materials). In the case of multiple publications from the same data set, the article having the most complete data was included. If partial data were reported in articles, then all such articles were included.

### 2.4 Assessment of methodological quality

The methodological quality of the included studies was assessed using the JBI checklist for observational studies by two independent reviewers (NG and IB).^41^, ^42^, ^43^ Any disagreements that arose between them were resolved through discussion. If a consensus was not reached, a third reviewer (KC) was involved. No cut‐off quality score was applied to exclude studies; therefore, all eligible studies regardless of their methodological quality were included in this review.

### 2.5 Data extraction

The data were extracted from included studies using a pre‐developed and piloted data extraction form. The following details were extracted: publication details (first author and year of publication), Indian state, study design, study year, study setting, population characteristics (study population ≥60 years only; sample size, mean age (in years), and number of females), unadjusted risk factors, adjusted risk factors (as reported by study authors), assessment of risk factors (e.g., self‐reported by participants or using medical records), definition of multimorbidity, and assessment of multimorbidity (e.g., self‐reported by participants or using medical records). Odds ratios (ORs) and 95% confidence intervals (CIs) were also extracted. Adjusted ORs were preferred over unadjusted ORs. In the absence of adjusted ORs, unadjusted ORs were extracted or calculated (using the available raw data). If a study had multiple categories for a risk factor, two or more categories were merged meaningfully to form a new category for meta‐analysis. For example, if general, scheduled castes, scheduled tribes, and other backward classes were the categories available for social caste, then the general category was considered as the reference group, and all the other categories were combined to form a new category. Two reviewers (NG and IB) independently extracted the data. Any disagreements that arose between them were resolved through discussion. If a consensus was not reached, a third reviewer (KC) was consulted.

### 2.6 Data synthesis

Initially, narrative synthesis was conducted. Where at least two studies reported the same or similar risk factors, a meta‐analysis was conducted using Review Manager (RevMan) 5.4 software.^44^ The ORs with 95% CIs were pooled using the random effects meta‐analysis approach and generic inverse variance.^45^, ^46^ The standard errors were used in creating the forest plots, which were calculated in STATA v17 using the following formula: standard error = (log upper CI–log lower CI)/3.92.^47^ The statistical heterogeneity across studies was estimated using I^2^ statistics. The I^2^ values of <50%, between 50% and 74%, and ≥75% were interpreted as low, moderate, and high levels of statistical heterogeneity, respectively.^48^

### 2.7 Assessment of publication bias

The publication bias was assessed using a funnel plot, provided at least 10 studies were included in the meta‐analysis.^49^

## 3 RESULTS

### 3.1 Inclusion of studies

**Figure 1:**
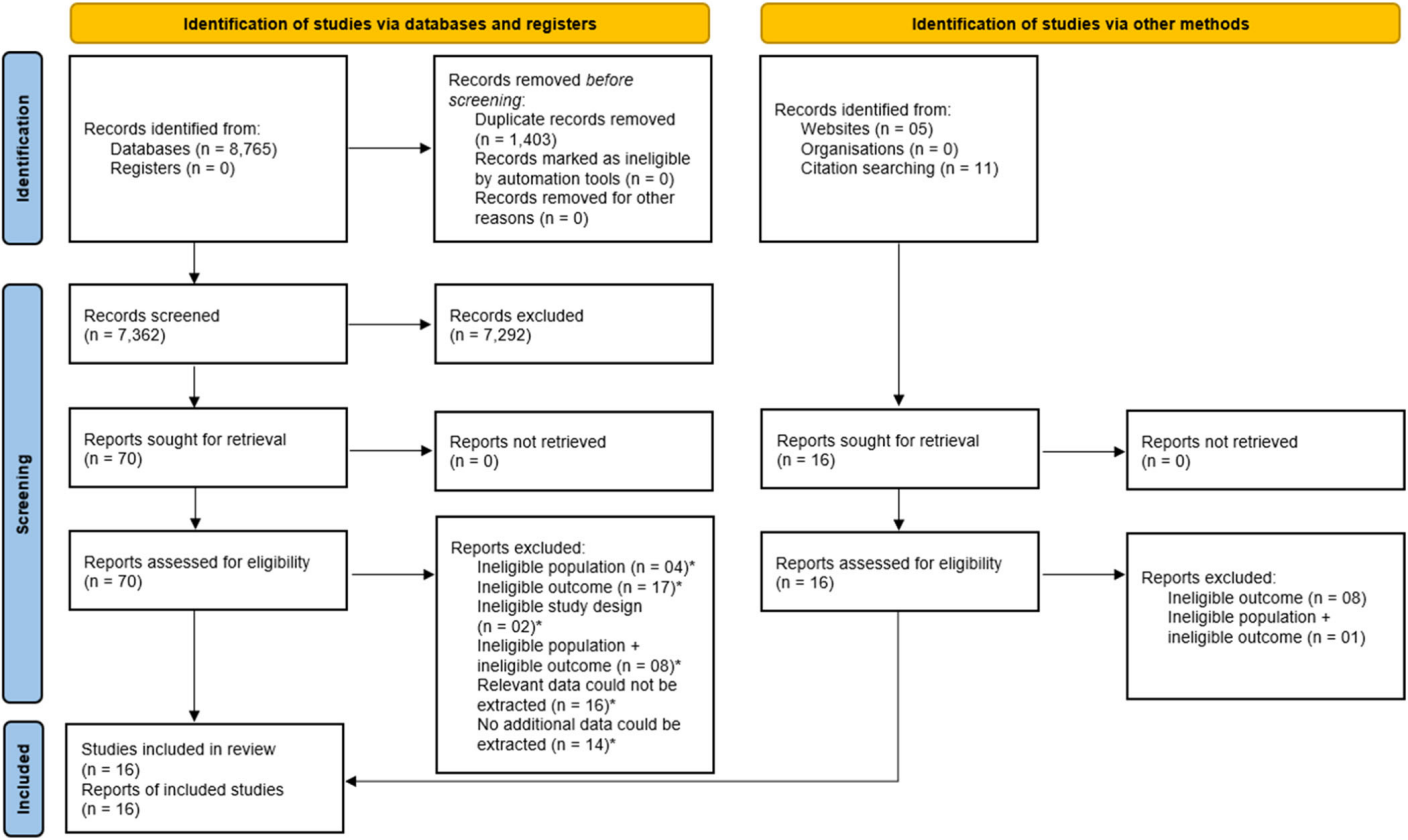
PRISMA flow diagram for systematic reviews which included searches of databases, registers, and other sources. *See Supplementary Materials.

Eight thousand seven hundred eighty‐one records were identified from the literature search, and all were in the English language. After the removal of duplicates, 7378 records were left for the title and abstract screening. Following the title and abstract screening, 93 records were left for the full‐text screening. Finally, 16 studies were included in this systematic review.^1^, ^18^, ^19^, ^50^, ^51^, ^52^, ^53^, ^54^, ^55^, ^56^, ^57^, ^58^, ^59^, ^60^, ^61^, ^62^ Out of these 16 studies, 15 were included in the meta‐analysis.^1^, ^18^, ^19^, ^50^, ^52^, ^53^, ^54^, ^55^, ^56^, ^57^, ^58^, ^59^, ^60^, ^61^, ^62^ Figure 1 shows the process of study selection and inclusion.

### 3.2 Characteristics of included studies

The characteristics of the included studies are presented in Table 1. Five studies were conducted on a nationally representative sample,^19^, ^50^, ^57^, ^61^, ^65^ five in the northern states,^18^, ^54^, ^55^, ^58^, ^60^ three in the eastern states,^1^, ^52^, ^53^ two in the southern states,^56^, ^62^ and one in both northern and southern states.^51^ All included studies were cross‐sectional and conducted in the community except for four (one each was conducted in residential care,^56^ primary care,^62^ both community and primary care,^18^ and community, residential care, primary care, secondary care, and tertiary care).^58^ The studies were published in 2004 and after. The sample size of older adults in the included studies varied from 148 to 42,756. The mean age of older adults in the included studies ranged from 66 to 75 years. Broadly, sociodemographic, lifestyle, and health conditions‐related factors were explored in the studies. All included studies used self‐reported data on exposures except for one (which used medical records).^62^ The same definition of multimorbidity (i.e., the coexistence of two or more chronic health conditions in an individual) was reported in 12 studies, whereas it was unclear in three studies,^53^, ^55^, ^60^ and in one study, no definition was provided.^50^ Nine studies used self‐reported data on multimorbidity,^1^, ^19^, ^51^, ^52^, ^56^, ^57^, ^58^, ^59^, ^61^ one used medical records,^62^ four used both self‐reported data and medical records,^18^, ^53^, ^54^, ^55^ one screened selected chronic health conditions,^60^ and one had not reported the details.^50^

**Table 1:** Characteristics of included studies.

First author and year of publication	Indian state	Study design	Study year	Study setting	Study population ≥60 years only?	Sample size (*n*)*	Mean age (in years)*	Females (*n*)*	Unadjusted risk factors°	Adjusted risk factors^	Assessment of risk factors	Definition of multimorbidity	Assessment of multimorbidity
Anushree 2022^50^	All states and all union territories	Cross‐sectional	2017‐2018	Community	Yes	42,756	Not reported	20,855	Gender (S), education (S), social caste (S), place of residence (S), wealth index (S)	Age (60–64 (ref), 65–70 (NS), ≥71 (S)), gender (NS), education (S), caste (S), place of residence (S), income (poorest (ref), poor (NS), middle (S), rich (S), richest (S))	Self‐reported by participants	Not defined	Self‐reported by participants
Arokiasamy 2015^51^	Karnataka, Kerala, Punjab, Rajasthan	Cross‐sectional	2010	Community	No	554	Not reported	Not extractable	Age (NS)	Not extractable	Self‐reported by participants	Simultaneous presence of two or more chronic conditions	Self‐reported by participants
Banjare 2014^52^	Odisha	Cross‐sectional	2011‐2012	Community	Yes	310	Not reported	157	Sex (S), age (S), marital status (NS), education (NS), social caste (NS), economic dependency (S), living arrangement (NS), smoking (NS), tobacco consumption (NS), wealth index (NS)	Sex (NS), age (S), marital status (NS), wealth index (NS), education (NS), caste (NS), state of economic independence (S), living arrangement (NS), smoking (S), chewing tobacco (S)	Self‐reported by participants	Having two or more morbidities	Self‐reported by participants
Chakraborty 2004^53^	West Bengal	Cross‐sectional	2004	Community	Yes	420	Not reported	261	Gender (NS), living arrangement (NS), age (S), marital status (S), attitude towards aging (healthy (ref), despair (S), non‐committal (NS))	Not reported	Self‐reported by participants	Not defined	Self‐reported by participants and their relatives, medical records
Jain 2016^54^	Punjab	Cross‐sectional	2014	Community	Yes	534	66.27	290	Age (S), gender (S), education (NS), marital status (S), socioeconomic status (S) body mass index (NS), family type (S)	Not reported	Self‐reported by participants	Any combination of chronic disease with at least one other disease (acute or chronic), or biopsychosocial factor (associated or not) or somatic risk factor	Self‐reported by participants, medical records
Kaur 2019^55^	City in North India	Cross‐sectional	2016	Community	Yes	225	70.60	115	Age (NS)	Not extractable	Self‐reported by participants	Not defined	Self‐reported by participants, medical records
Kshatri 2020^1^	Odisha	Cross‐sectional	2019‐2020	Community	Yes	725	70.24	347	Age (S), gender (NS), family type (NS), education (NS), work status (S), socioeconomic status (NS), social caste (S), smoking (NS), tobacco consumption (NS), alcohol consumption (S)	Current smoking (NS), smokeless tobacco (NS), alcohol consumption (NS), family history of diabetes (S), family history of hypertension (S)	Self‐reported by participants	Co‐occurrence of multiple chronic conditions without taking any of the diseases as the index condition	Self‐reported by participants
Maramula 2022^56^	Telangana	Cross‐sectional	2017‐2019	Residential care	Yes	1,182	75	764	Age (NS), gender (NS), education (NS), smoking (NS), alcohol consumption (NS), body mass index (S)	Age (60–69 (ref), 70–79 (S), 80 and above (NS)), gender (NS), education (NS), type of home (S), smoking status (S), alcohol consumption (NS), body mass index (S)	Self‐reported by participants	Presence of two or more systemic health issues (non‐communicable diseases)	Self‐reported by participants
Muhammad 2022,^57^ Chauhan 2022^19^	All states (except Sikkim) and all union territories	Cross‐sectional	2017‐2018	Community	Yes	31,464 (Muhammad 2022)/31,373 (Chauhan 2022)	Not reported	16,366 (Muhammad 2022)/16,565 (Chauhan 2022)	Body mass index (S), age (S), gender (S), education (S), work status (S), tobacco consumption (S), alcohol consumption (S), wealth index (S), religion (S), social caste (S), place of residence (S), geographical region (NS), living arrangement (NS)	Obese/overweight (S), high risk waist circumference (S), high risk waist‐hip ratio (S), age (young‐old (ref), old‐old (S), oldest‐old (NS)), education (S), marital status (currently married (ref), widowed (NS), others (S)), working status (S), tobacco consumption (NS), alcohol consumption (NS), physical activity (frequently (ref), rare (NS), never (S)), monthly per capita consumption expenditure quintile (S), religion (Hindu (ref), Muslim (S), Christian (S), others (NS)), caste (scheduled caste (ref), scheduled tribe (S), other backward class (NS), others (S)), place of residence (S), region (S), living arrangement (NS), self‐rated health (S), activities of daily living (ADL) disability (severe ADL (ref), moderate ADL(NS), no ADL (S)), independent activities of daily living (IADL) (severe IADL (ref), moderate IADL (S), no IADL (NS))	Self‐reported by participants	Presence of two or more chronic diseases	Self‐reported by participants
Nanda 2020^58^	Jammu and Kashmir	Cross‐sectional	2017‐2019	Community, residential care, primary care, secondary care, tertiary care	Yes	750	Not reported	358	Gender (NS), age (S), place of residence (S), religion (S), family type (NS), economic dependency (S), socioeconomic status (NS)	Gender (NS), age (60‐64 (ref),65–69 (S), 70–74 (S), 75‐79 (NS), 80‐84 (NS), 85 and above (NS)), area (NS), religion (Hindu (ref), Muslim (S), Sikh (NS)), family type (NS), dependency status (NS), socioeconomic status (NS)	Self‐reported by participants	Simultaneous occurrence of several adverse conditions to the same person	Not reported
Pati 2014^59^	Assam, Karnataka, Maharashtra, Rajasthan, Uttar Pradesh, West Bengal	Cross‐sectional	2007	Community	No	3,543	Not reported	Not extractable	Age (S)	Not extractable	Self‐reported by participants	Presence of two or more noncommunicable diseases	Self‐reported by participants
Prabhakar 2021^60^	Delhi	Cross‐sectional	2019	Community	Yes	350	68.26	191	Gender (S)	Not reported	Self‐reported by participants	Not defined	Screened selected chronic health conditions
Sharma 2022^61^	Himachal Pradesh, Punjab, West Bengal, Odisha, Maharashtra, Kerala, Tamil Nadu	Cross‐sectional	2011	Community	Yes	9,852	Not reported	5180	Gender (S), age (S), education (NS), place of residence (NS), marital status (S), social caste (S), religion (S), living arrangement (S), work status (S), wealth index (S), smoking (S), tobacco consumption (NS), alcohol consumption (S), geographical region (S)	Gender (S), age (S), educational attainment (no education (ref), 1–5 years (S), 6–9 years (NS), 10+ years (NS)), place of residence (NS), marital status (NS), caste (NS), religion (S), living arrangement (NS), current working status (S), wealth quintile (poorest (ref), poor (NS), middle (S), rich (NS), richest (S)), smoking (S), tobacco consumption (NS), alcohol consumption (S), state (S)	Self‐reported by participants	Two or more chronic conditions simultaneously	Self‐reported by participants
Vargese 2020^62^	Kerala	Cross‐sectional	2017	Primary care	No	148	Not reported	Not extractable	Age (NS)	Not extractable	Medical records	Co‐occurrence of two or more chronic conditions	Medical records
Verma 2019^17^	Uttar Pradesh	Cross‐sectional	2014‐2015	Community, primary care	Yes	400	68.86	185	Gender (S), age (NS), education (S), marital status (NS), socioeconomic status (NS)	Not reported	Self‐reported by participants	Two or more chronic conditions	Self‐reported by participants, medical records

* ≥60 years only.

^°^ Significant (S)/nonsignificant (NS) as calculated by reviewers.

^^^ Significant (S)/nonsignificant (NS) as reported by study authors.

### 3.3 Methodological quality of included studies

The methodological quality of the included studies is presented in Table 2. The critical appraisal scores varied from 38% to 88%. Ten studies obtained a score of more than 70%, that is, answered ‘yes’ to at least six questions on the JBI checklist for analytical cross‐sectional studies.^1^, ^19^, ^50^, ^51^, ^56^, ^57^, ^58^, ^59^, ^61^, ^62^ The inclusion criteria were clearly defined in all the studies except for one.^52^ All included studies described study participants and settings in detail except for one (where the exact study location was unclear).^55^ In nine studies, exposure was not measured using a valid and reliable method.^18^, ^19^, ^50^, ^52^, ^54^, ^56^, ^60^, ^61^, ^62^ The definition of multimorbidity was unclear in three studies,^53^, ^55^, ^60^ and one had not defined it.^50^ Two studies did not identify the confounders,^53^, ^54^ and four had not stated strategies to deal with them.^18^, ^52^, ^54^, ^60^ Only two studies assessed multimorbidity using a reliable and valid method.^53^, ^60^ Appropriate statistical analysis, such as multivariable logistic regression, was used in all included studies except for four.^18^, ^53^, ^54^, ^60^

**Table 2:** Methodological quality of included studies.

Study	Q1	Q2	Q3	Q4	Q5	Q6	Q7	Q8	Critical appraisal score (total % of “yes” to critical appraisal questions)
Anushree 2022^50^	Y	Y	Y	N	Y	Y	N	Y	75
Arokiasamy 2015^51^	Y	Y	U	Y	Y	Y	U	Y	75
Banjare 2016^52^	U	Y	U	Y	Y	Y	U	Y	63
Chakraborty 2004^53^	Y	Y	Y	U	U	N	Y	U	50
Chauhan 2022^19^	Y	Y	U	Y	Y	Y	N	Y	75
Jain 2016^54^	Y	Y	U	Y	U	N	U	N	38
Kaur 2019^55^	Y	U	Y	U	Y	Y	U	Y	63
Kshatri 2020^1^	Y	Y	Y	Y	Y	Y	U	Y	88
Maramula 2020^56^	Y	Y	U	Y	Y	Y	N	Y	75
Muhammad 2022^57^	Y	Y	Y	Y	Y	Y	N	Y	88
Nanda 2020^58^	Y	Y	Y	Y	Y	Y	N	Y	88
Pati 2014^59^	Y	Y	Y	Y	Y	Y	N	Y	88
Prabhakar 2021^60^	Y	Y	U	U	Y	N	Y	N	50
Sharma 2022^61^	Y	Y	U	Y	Y	Y	N	Y	75
Vargese 2020^62^	Y	Y	U	Y	Y	Y	N	Y	75
Verma 2019^18^	Y	Y	U	Y	Y	N	Y	N	63
Total % of “yes” to each critical appraisal question	94	94	44	75	88	75	19	75	

*Note*: 1. Were the criteria for inclusion in the sample clearly defined? 2. Were the study subjects and the setting described in detail? 3. Was the exposure measured in a valid and reliable way? 4. Were objective, standard criteria used for measurement of the condition? 5. Were confounding factors identified? 6. Were strategies to deal with confounding factors stated? 7. Were the outcomes measured in a valid and reliable way? 8. Was appropriate statistical analysis used?

Abbreviations: N, no; U, unclear; Y, yes.

### 3.4 Meta‐analysis

Fifteen studies were included in the meta‐analysis.

#### 3.4.1 Sociodemographic factors

1. Age

**Figure 2:**
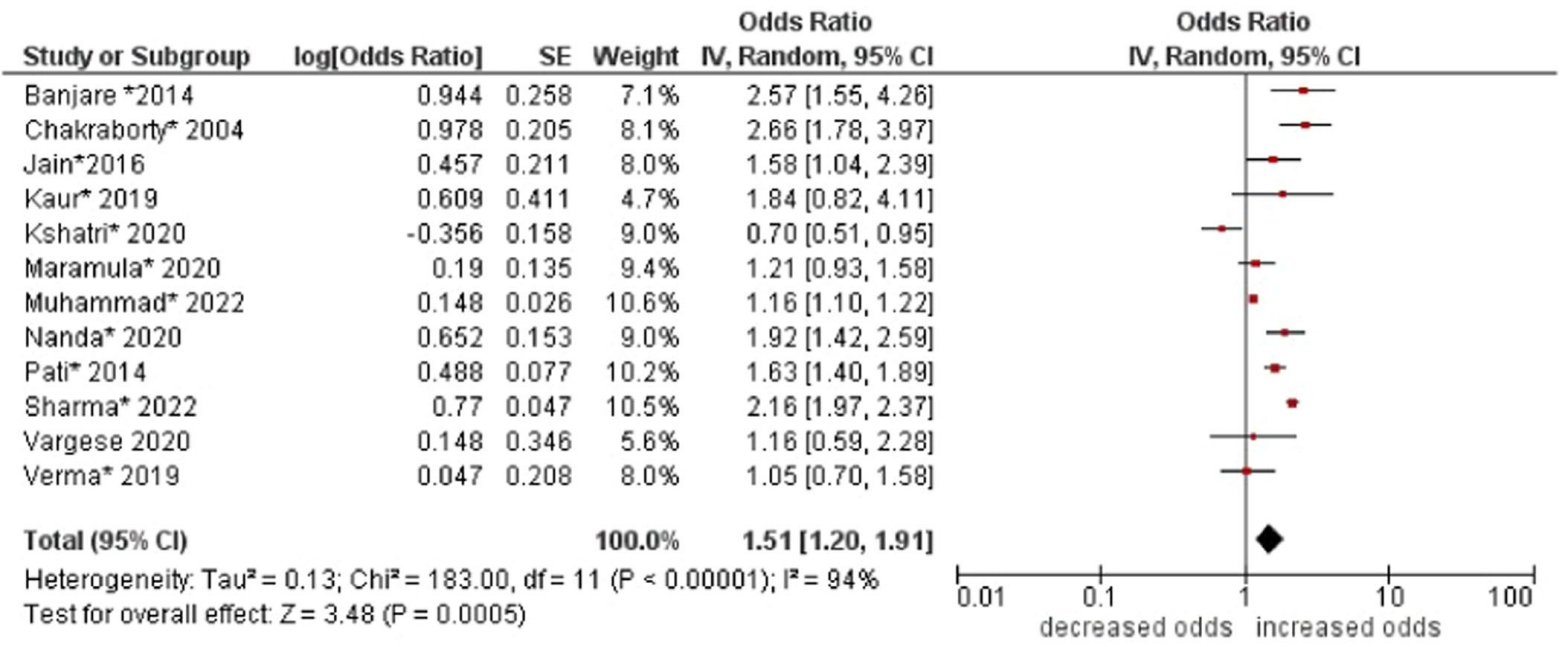
Forest plot of the association between age and multimorbidity. 60–69 years of age was the reference group, and ≥70 years was the other group. *Unadjusted ORs and 95% CI.

The pooled odds of multimorbidity were higher in people aged ≥70 years compared to those aged 60–69 years (OR: 1.51; 95% CI: 1.20–1.91). High statistical heterogeneity was found across studies (I^2^ 94%) (see Figure 2).

2. Sex

**Figure 3:**
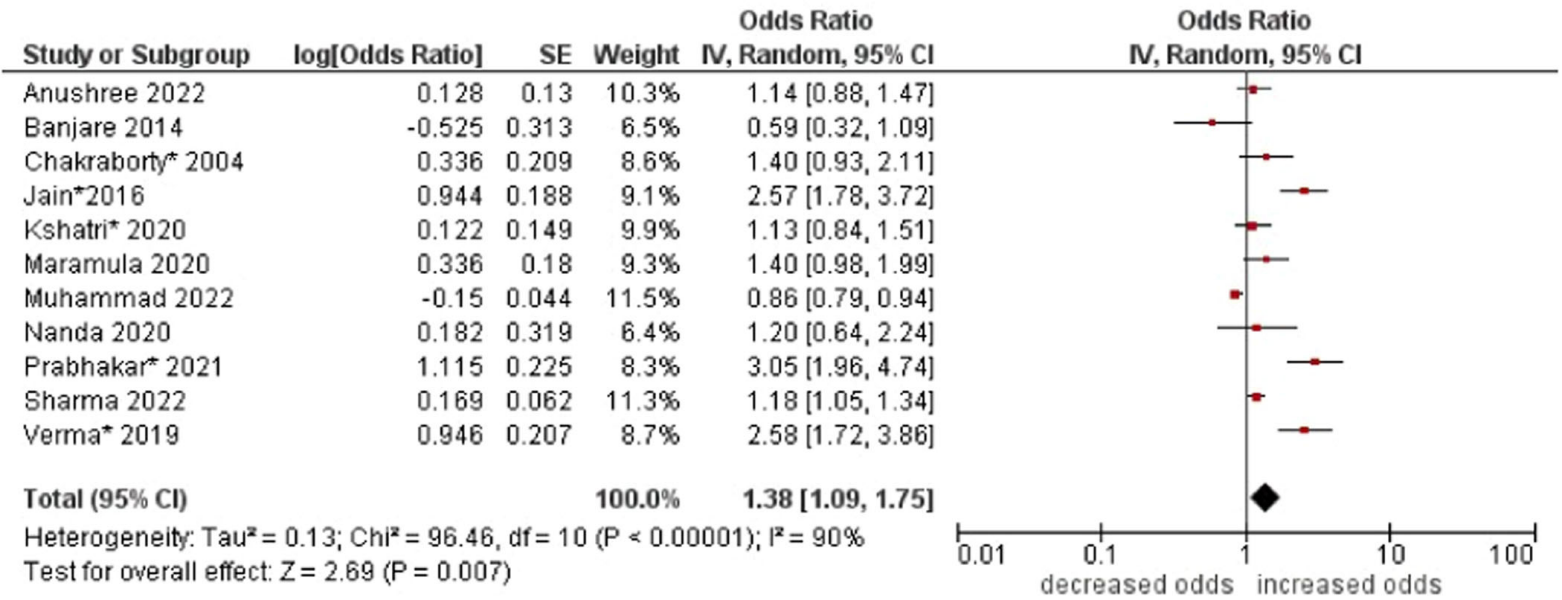
Forest plot of the association between sex and multimorbidity. Studies reporting either sex or gender were combined as ‘sex’. Male was the reference group, and female was the other group. *Unadjusted ORs and 95% CI.

The pooled odds of multimorbidity were higher in females compared to males (OR: 1.38; 95% CI: 1.09–1.75). High statistical heterogeneity was found across studies (I^2^ 90%) (see Figure 3).

3. Social caste

**Figure 4:**
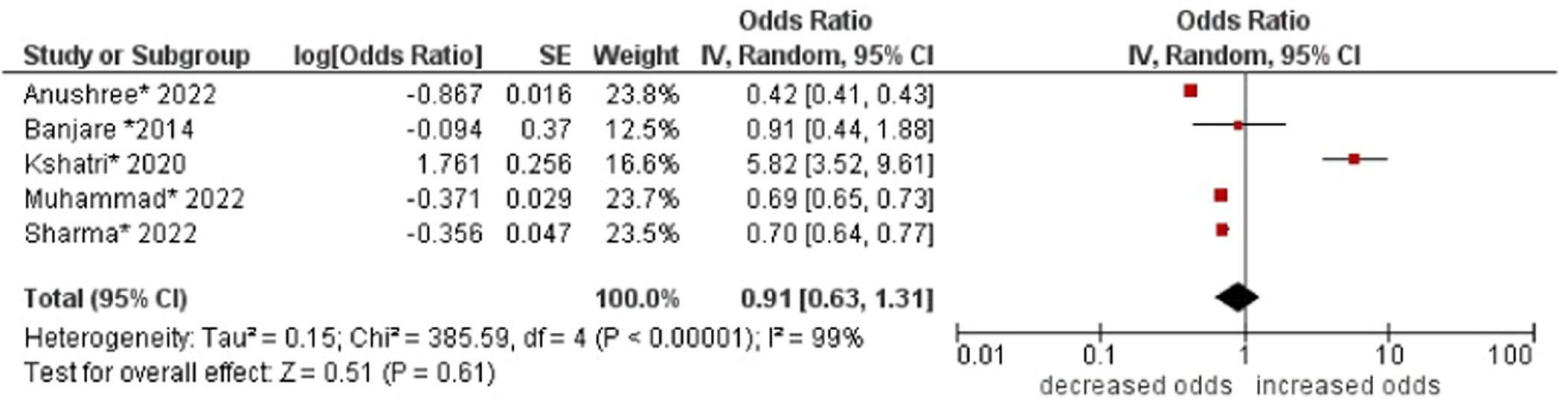
Forest plot of the association between social caste and multimorbidity. The general category of social caste was the reference group, and other categories (i.e., scheduled castes, scheduled tribes, and other backward classes) were combined to form the other group. *Unadjusted ORs and 95% CI.

No association was found between social caste and multimorbidity (OR: 0.91; 95% CI: 0.63–1.31). High statistical heterogeneity was found across studies (I^2^ 99%) (see Figure 4).

4. Religion

**Figure 5:**
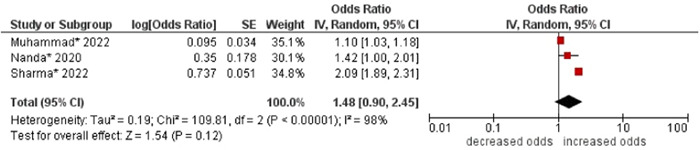
Forest plot of the association between religion and multimorbidity. Hindu religion was the reference group, and other categories (i.e., Muslims, Sikhs, Christians, and others) were combined to form the other group. *Unadjusted ORs and 95% CI.

No association was found between religion and multimorbidity (OR: 1.48; 95% CI 0.90–2.45). High statistical heterogeneity was found across studies (I^2^ 98%) (see Figure 5).

5. Education

**Figure 6:**
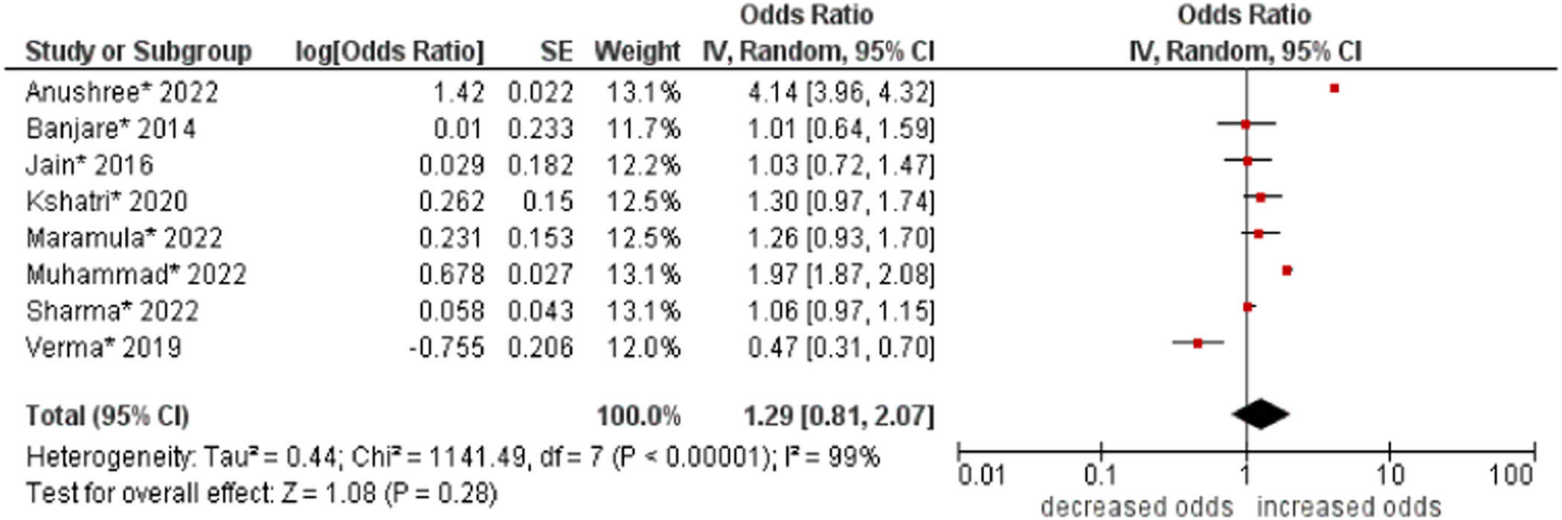
Forest plot of the association between education and multimorbidity. No education or illiteracy was combined as the reference group, and primary school, secondary school, and higher education were combined to form the other group. *Unadjusted ORs and 95% CI.

No significant association was found between education and multimorbidity (OR: 1.29; 95% CI: 0.81–2.07). High statistical heterogeneity was found across studies (I^2^ 99%) (see Figure 6).

6. Marital status

**Figure 7:**
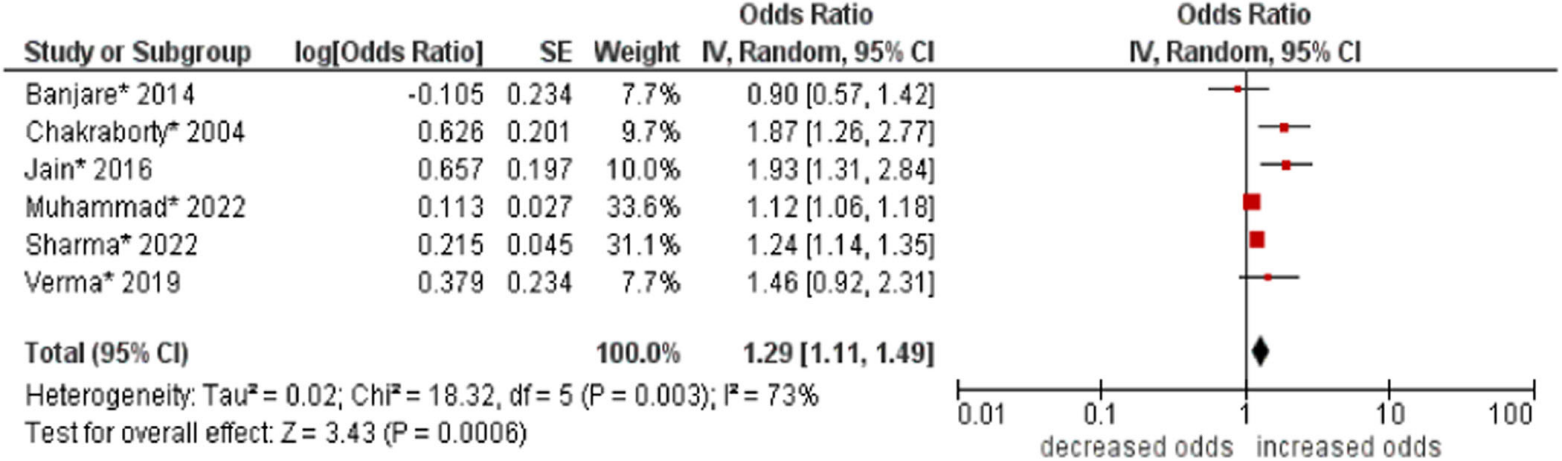
Forest plot of the association between marital status and multimorbidity. Married was the reference group, and other categories (i.e., single, divorced, separated, and widowed) were combined to form the other group. *Unadjusted ORs and 95% CI.

The pooled odds of multimorbidity were higher in single, divorced, separated, and widowed people compared to married people (OR: 1.29; 95% CI: 1.11–1.49). Moderate statistical heterogeneity was found across studies (I^2^ 73%) (see Figure 7).

7. Family type

**Figure 8:**
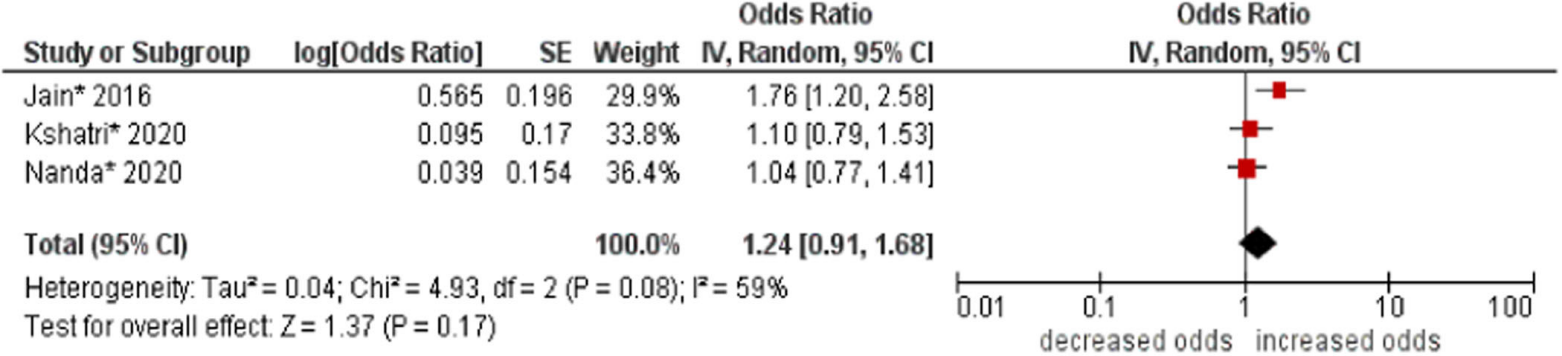
Forest plot of the association between family type and multimorbidity. The nuclear family was the reference group, and the joint family was the other group. *Unadjusted ORs and 95% CI.

No association was found between family type and multimorbidity (OR: 1.24, 95% CI: 0.91–1.68). Moderate statistical heterogeneity was found across studies (I^2^ 59%) (see Figure 8).

8. Living arrangements

**Figure 9:**
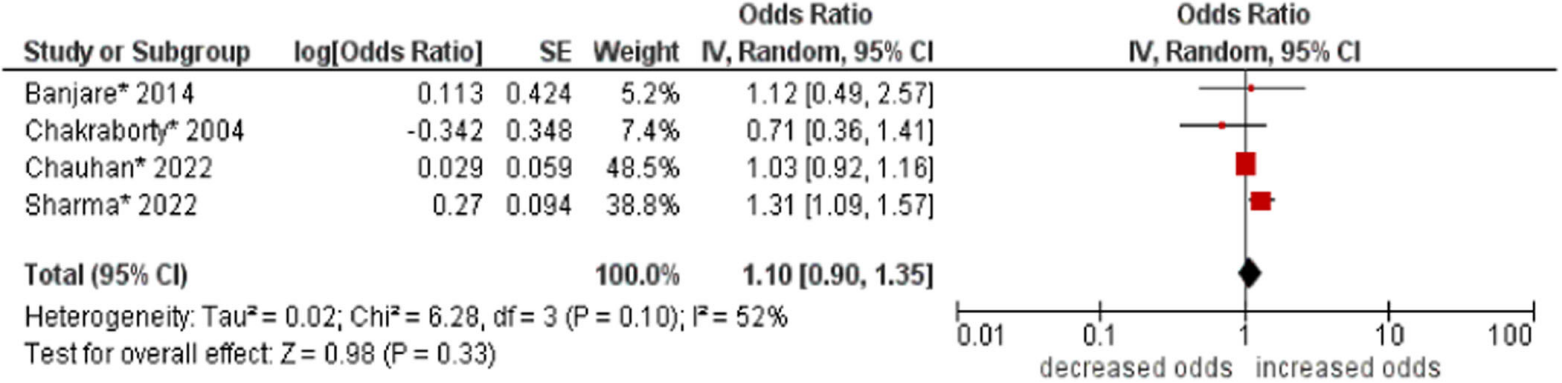
Forest plot of the association between living arrangements and multimorbidity. Living alone was the reference group, and other categories (i.e., living with a spouse, living with children, living with both spouse and children, and living with others) were combined to form the other group. *Unadjusted ORs and 95% CI.

No association was found between living arrangements and multimorbidity (OR: 1.10; 95% CI: 0.90–1.35). Moderate statistical heterogeneity was found across studies (I^2^ 52%) (see Figure 9).

9. Economic dependency

**Figure 10:**
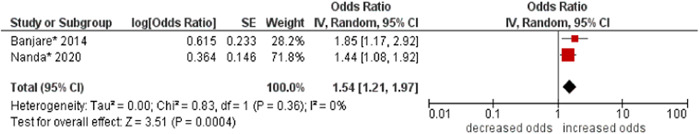
Forest plot of the association between economic dependency and multimorbidity. Economically independent was the reference group, and partially dependent and totally dependent were combined to form the other group (i.e., economically dependent). *Unadjusted ORs and 95% CI.

The pooled odds of multimorbidity were higher in economically dependent people compared to economically independent people (OR: 1.54; 95% CI: 1.21–1.97). No statistical heterogeneity was found across studies (I^2^ 0%) (see Figure 10).

10. Work status

**Figure 11:**
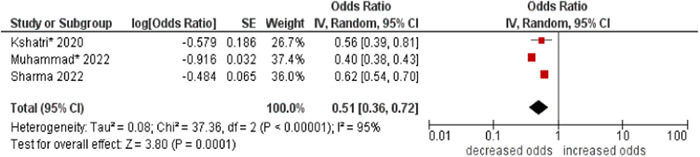
Forest plot of the association between work status and multimorbidity. Not working was the reference group, and working was the other group. *Unadjusted ORs and 95% CI.

The pooled odds of multimorbidity were lower in working people compared to those not working (OR: 0.51; 95% CI: 0.36–0.72). High statistical heterogeneity was found across studies (I^2^ 95%) (see Figure 11).

11. Socioeconomic status

**Figure 12:**
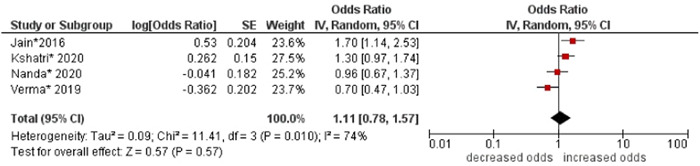
Forest plot of the association between socioeconomic status and multimorbidity. Lower categories of socioeconomic status (i.e., low, lower, and upper lower) were combined as the reference group, and higher categories of socioeconomic status (i.e., middle and upper) were combined to form the other group. *Unadjusted ORs and 95% CI.

No association was found between socioeconomic status and multimorbidity (OR: 1.11; 95% CI: 0.78–1.57). Moderate statistical heterogeneity was found across studies (I^2^ 74%) (see Figure 12).

12. Wealth index

**Figure 13:**
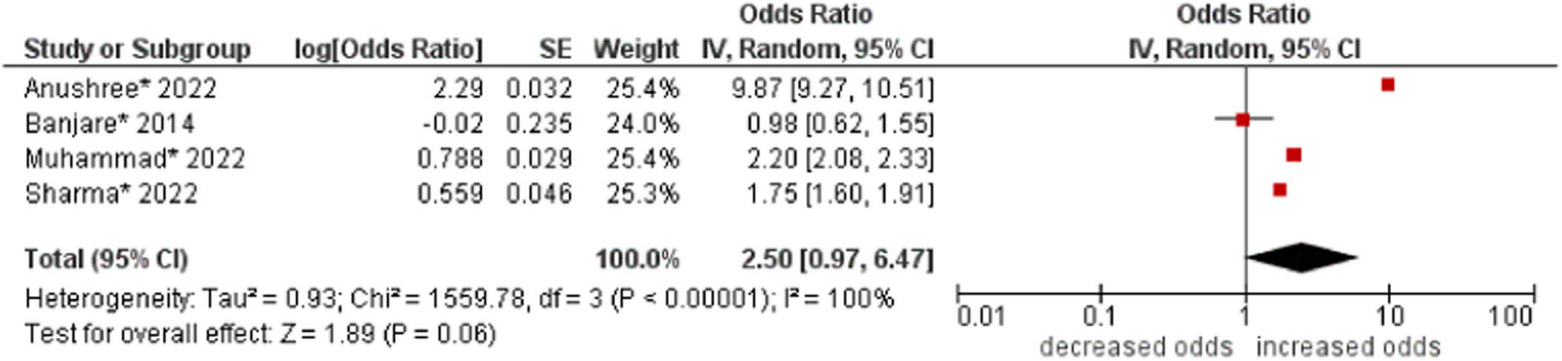
Forest plot of the association between wealth index and multimorbidity. Lower categories of wealth index (i.e., poor, poorer, and poorest) were combined as the reference group, and higher categories of wealth index (i.e., middle and upper) were combined to form the other group. *Unadjusted ORs and 95% CI.

No association (borderline) was found between wealth index and multimorbidity (OR: 2.50; 95% CI: 0.97–6.47). High statistical heterogeneity was found across studies (I^2^ 100%) (see Figure 13).

13. Place of residence

**Figure 14:**
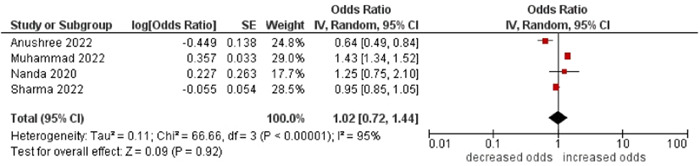
Forest plot of the association between place of residence and multimorbidity. The rural area was the reference group, and the urban area was the other group. *Unadjusted ORs and 95% CI.

No association was found between place of residence and multimorbidity (OR: 1.02; 95% CI: 0.72–1.44). High statistical heterogeneity was found across studies (I^2^ 95%) (see Figure 14).

14. Geographical region

**Figure 15:**
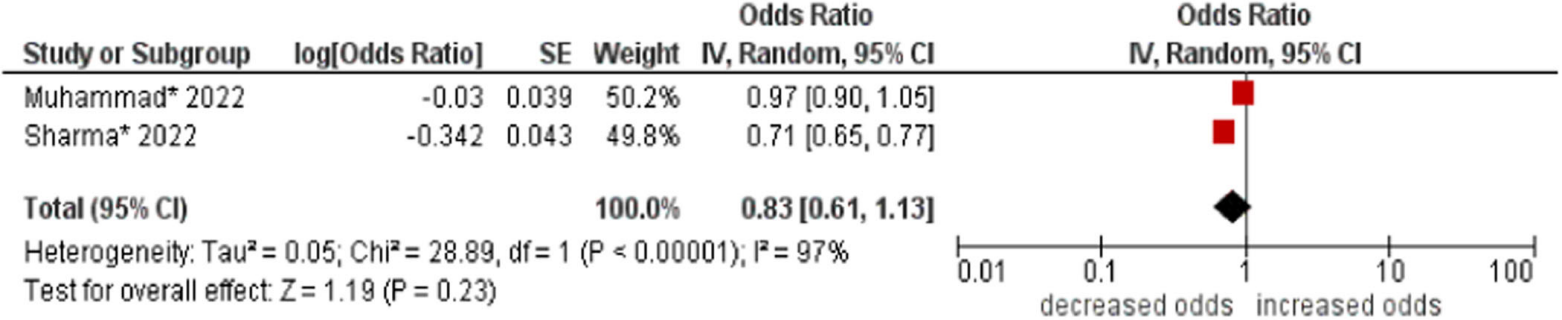
Forest plot of the association between geographical region and multimorbidity. Northern India was the reference group, and other regions in India were combined as the other group. *Unadjusted ORs and 95% CI.

No association was found between geographical region and multimorbidity (OR: 0.83; 95% CI: 0.61–1.13). High statistical heterogeneity was found across studies (I^2^ 97%) (see Figure 15).

#### 3.4.2 Lifestyle factors

1. Smoking

**Figure 16:**
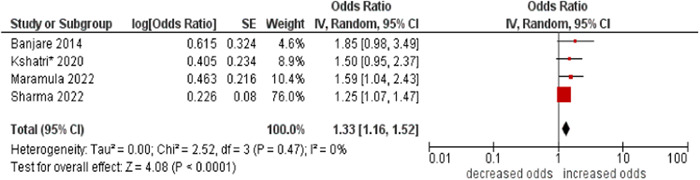
Forest plot of the association between smoking and multimorbidity. No smoking was the reference category, and smoking was the other group. *Unadjusted ORs and 95% CI.

The pooled odds were higher in smokers compared to non‐smokers (OR: 1.33; 95% CI: 1.16–1.52). No statistical heterogeneity was found across studies (I^2^ 0%) (see Figure 16).

2. Tobacco consumption

**Figure 17:**
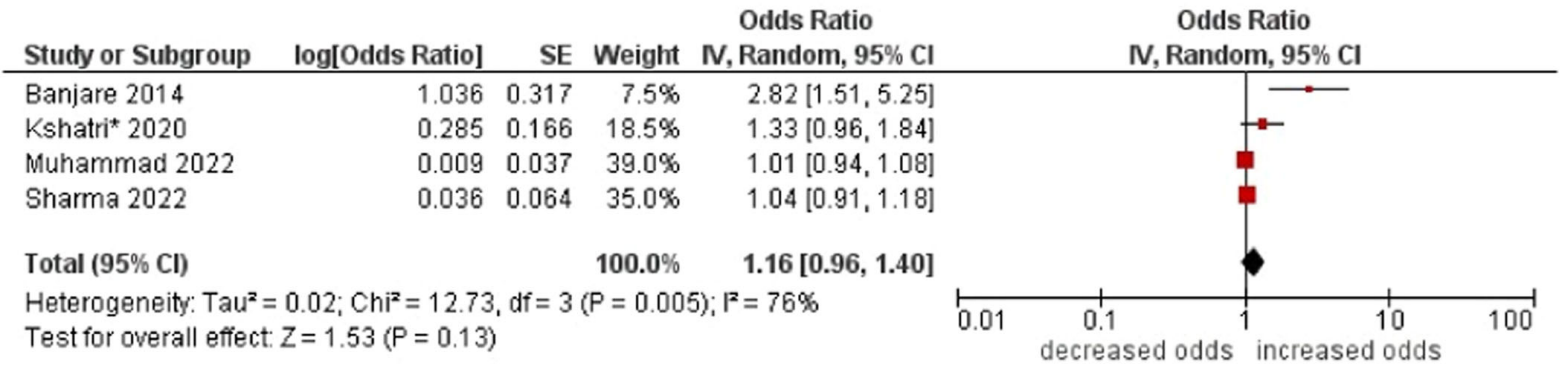
Forest plot of the association between tobacco consumption and multimorbidity. No tobacco consumption was the reference group, and tobacco consumption was the other group. *Unadjusted ORs and 95% CI.

No association was found between tobacco consumption and multimorbidity (OR: 1.16; 95% CI: 0.96–1.40). High statistical heterogeneity was found across studies (I^2^ 76%) (see Figure 17).

3. Alcohol consumption

**Figure 18:**
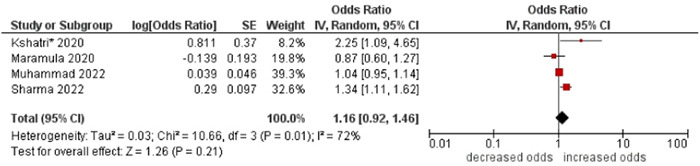
Forest plot of the association between alcohol consumption and multimorbidity. No alcohol consumption was the reference group, and alcohol consumption was the other group. *Unadjusted ORs and 95% CI.

No association was found between alcohol consumption and multimorbidity (OR: 1.16; 95% CI: 0.92–1.46). High statistical heterogeneity was found across studies (I^2^ 72%) (see Figure 18).

#### 3.4.3 Health conditions‐related factors

1. BMI

**Figure 19:**
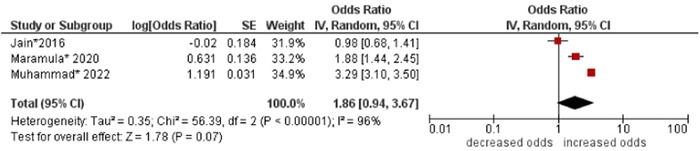
Forest plot of the association between BMI and multimorbidity. Lower two categories of BMI (i.e., underweight and normal) were combined as the reference group, and higher two categories of BMI (i.e., overweight and obesity) were combined to form the other group. *Unadjusted ORs and 95% CI.

No association was found between body mass index (BMI) and multimorbidity (OR: 1.86; 95% CI: 0.94–3.67). High statistical heterogeneity was found across studies (I^2^ 96%) (see Figure 19).

### 3.5 Publication bias

**Figure 20:**
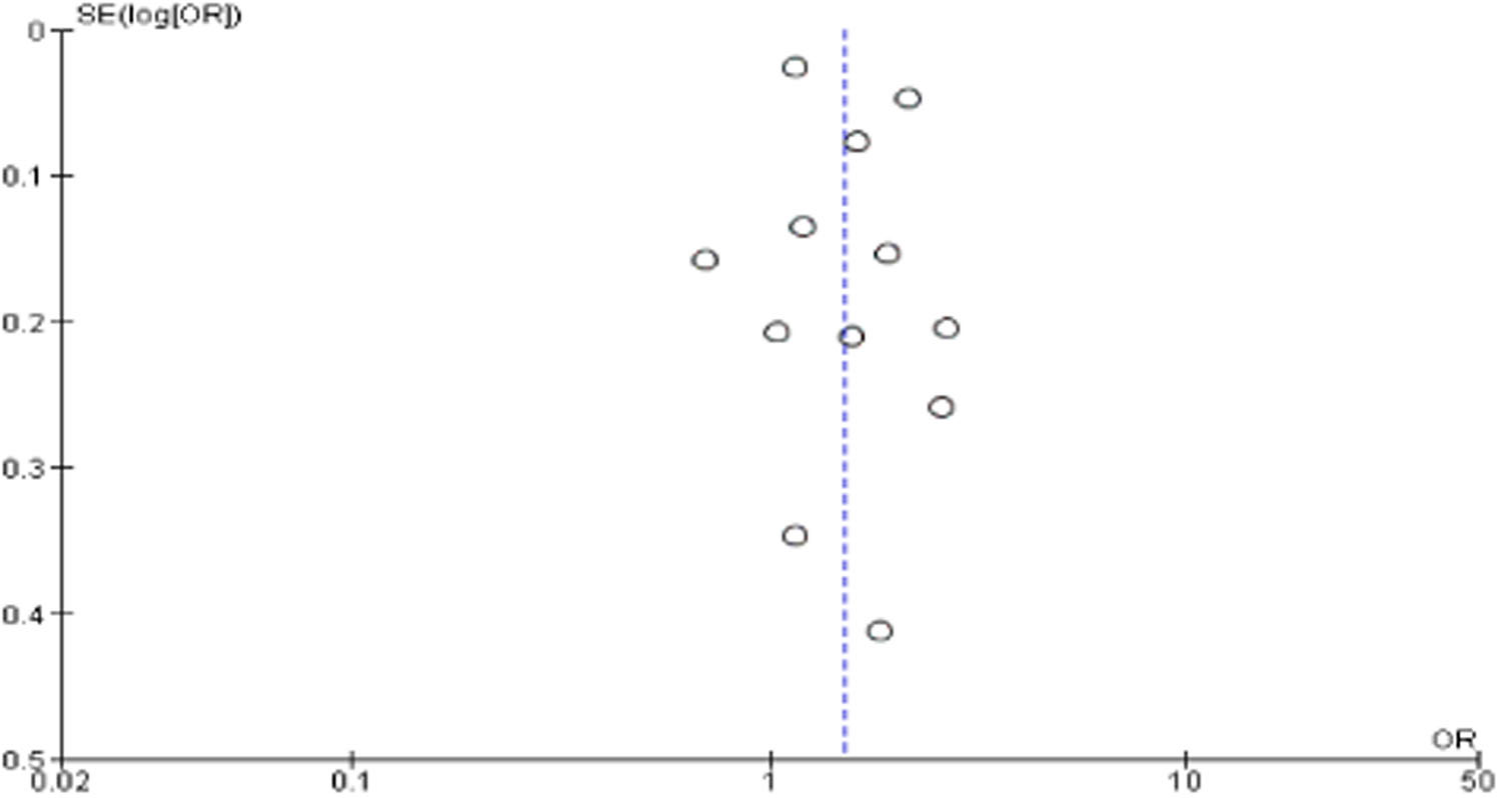
Funnel plot to assess publication bias for age as a risk factor.

**Figure 21:**
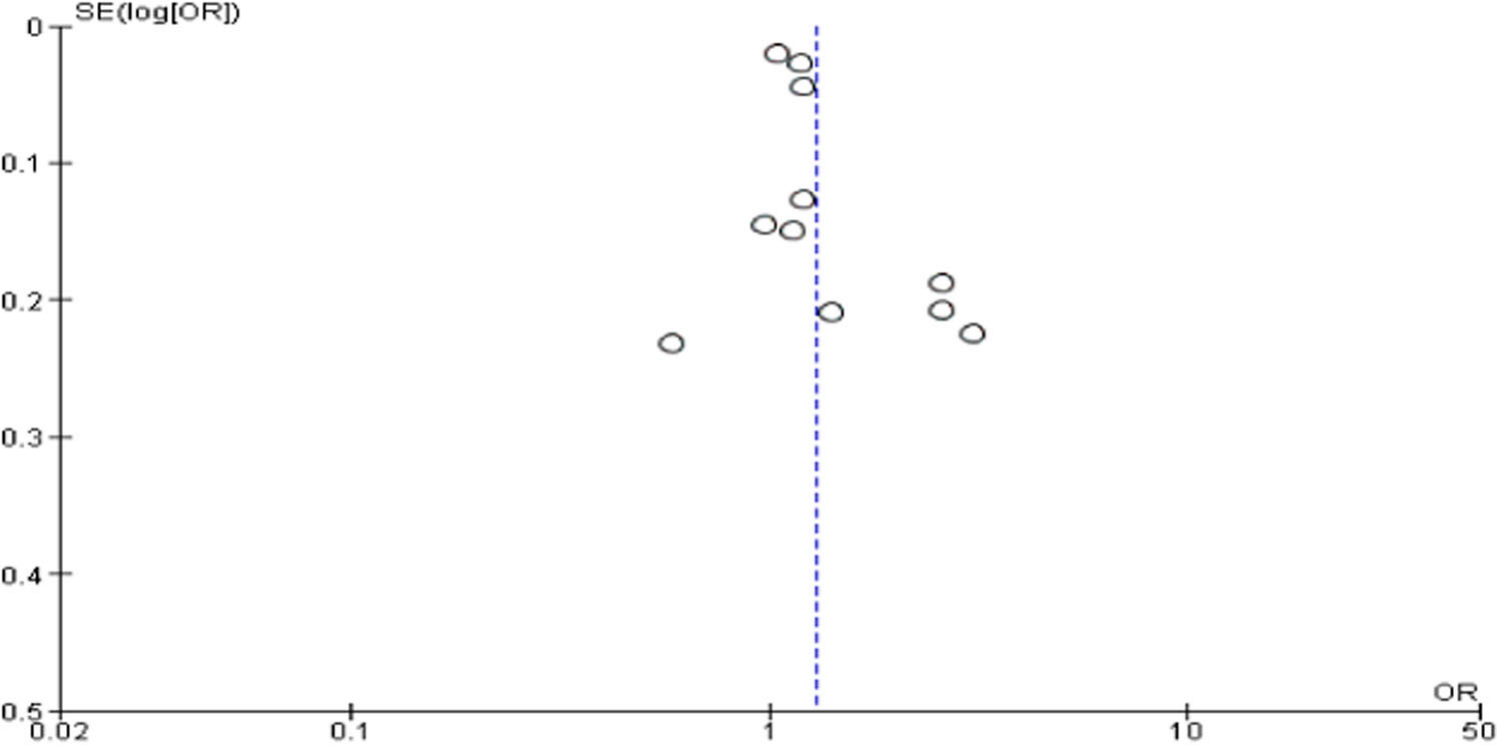
Funnel plot to assess publication bias for sex as a risk factor.

Publication bias was detected in the funnel plot for age but not for sex as a risk factor (see Figure 20 and Figure 21).

## 4 DISCUSSION

We conducted a systematic review and meta‐analysis on risk factors of multimorbidity among older adults in India. Broadly, sociodemographic, lifestyle, and health conditions‐related factors were explored in the included studies. The pooled odds of multimorbidity were higher in people aged ≥70 years, females, single, divorced, separated, and widowed, economically dependent, not working, and smokers.

In our review, higher age was found to be associated with multimorbidity. This finding is consistent with systematic reviews of studies conducted globally.^5^, ^14^, ^16^, ^37^, ^63^ Aging is a universal process that is accompanied by a decline in anatomical, immunological, and cognitive functions as a result of changes at the cellular level.^64^, ^65^ As individuals age, the number of chronic conditions, their severity, and associated adverse consequences like disability, become more profound and complex.^12^, ^13^ In our review, female sex was found to be associated with multimorbidity, which is consistent with available global evidence.^16^, ^63^, ^66^ A possible explanation could be inadequate access and utilization of healthy lifestyle practices and healthcare facilities for females in India^67^, ^68^ due to factors like socio‐cultural issues or personal choices.^67^ Females usually have a higher life expectancy than males^16^, ^67^ as they are more likely to suffer from nonfatal diseases.^16^ In this review, single, divorced, separated, and widowed people had higher odds of multimorbidity. The finding is in line with a cross‐sectional study conducted in Nepal, a neighboring country.^69^ Generally, married people tend to have better physical and mental health due to the emotional and financial support they receive from their partner.^69^, ^70^ However, factors like relationship quality and length could be also important. In our review, economic dependency was found to be associated with multimorbidity. Economic dependency can deprive individuals of a healthy lifestyle and receive high‐quality healthcare, which could explain the above‐mentioned association.^71^ Similarly, in this review, people who were not working had higher odds of multimorbidity. This is consistent with a systematic review of studies conducted in the WHO Eastern Mediterranean countries.^72^ Those who do not work can struggle with finances as well as physical and mental health.^72^, ^73^ In our review, smoking was found to be associated with multimorbidity. This is consistent with another systematic review of studies conducted globally.^37^ Smoking is an unhealthy lifestyle that predisposes individuals to the development of several health conditions.^74^ The cumulative toxic effects of smoking have a detrimental impact on health, particularly on the respiratory and cardiovascular systems.^75^ In other words, these synthesized sociodemographic and lifestyle factors should be taken into consideration when developing health interventions for addressing multimorbidity among older adults in India.

To the best of our knowledge, this is the first systematic review to synthesize the existing evidence on risk factors of multimorbidity among older adults in India. A robust systematic review process was followed, and several databases were searched for published and unpublished studies without any date and language restrictions and using comprehensive search strategies. Although a standardized critical appraisal tool was used in this review, the assessment of methodological quality is subjective to a large extent. However, the inter‐rater reliability was 96%. In the absence of adjusted ORs, unadjusted ORs were used in the meta‐analysis. The included studies were geographically well‐distributed across different states of India. Some of the studies were conducted on a nationally representative sample with a large sample size thus, giving a largely complete picture. In terms of generalizability, the findings could be valid in similar populations, settings, and contexts. To update this systematic review in the future, more primary studies should be conducted on other potential risk factors. For example, several factors (e.g., family history of diabetes, family history of hypertension, and level of physical activity) could not be included in any meta‐analysis due to being reported in single studies.^1^, ^18^, ^52^, ^55^, ^57^, ^61^ In addition, none of the included studies explored genetic and environmental factors. The included studies were all cross‐sectional, and thus, there is a need to conduct longitudinal studies to explore causality. Rather than relying completely on self‐reported data in primary studies, exposures and outcomes should be measured objectively. For example, screening people to identify chronic health conditions and reviewing medical records for medical and surgical history.

In conclusion, this systematic review and meta‐analysis provided a comprehensive picture of the problem by synthesizing the existing evidence on risk factors of multimorbidity among older adults in India. These synthesized sociodemographic and lifestyle factors should be taken into consideration when developing health interventions for addressing multimorbidity among older adults in India, and more specifically, in people aged ≥70 years, females, single, divorced, separated, and widowed, economically dependent and not working, and smokers.

## AUTHOR CONTRIBUTIONS

**Nikita Goel**: Conceptualization; data curation; formal analysis; investigation; methodology; project administration; resources; software; validation; visualization; writing—original draft; writing—review and editing. **Isha Biswas**: Data curation; formal analysis; methodology; software; validation; writing—review and editing. **Kaushik Chattopadhyay**: Conceptualization; investigation; methodology; resources; software; supervision; validation; visualization; writing—original draft; writing—review and editing.

## CONFLICT OF INTEREST STATEMENT

The authors declare no conflict of interest.

## TRANSPARENCY STATEMENT

The lead author Kaushik Chattopadhyay affirms that this manuscript is an honest, accurate, and transparent account of the study being reported; that no important aspects of the study have been omitted; and that any discrepancies from the study as planned (and, if relevant, registered) have been explained.

## ETHICS STATEMENT

Institutional Review Board approval was not required.

## Supporting information

Supporting information.

Supporting information.

## Data Availability

The authors confirm that the original data supporting the findings of this study are included in the article and supplementary materials.
