# Supplementary material for: Risk factors of multimorbidity among older adults in India: A systematic review and meta‐analysis
Source: Health Sci Rep. 2024 Feb 28;7(2):e1915. doi: 10.1002/hsr2.1915 (PMC10900089; doi:10.1002/hsr2.1915)
Supplement: Supplementary file 1 — Supporting information. [file HSR2-7-e1915-s002.docx]

**Supplementary Material S1: Search Strategy**

**MEDLINE (OVID) (1946- August 03, 2022): 3180 records**

1. Exp risk factors/
2. (Risk factor* or risk-factor*).mp
3. (Risk assess* or risk-assess*).mp
4. “Risk analys#s”.mp
5. Determinant*.mp
6. Predict*.mp
7. Risk*.mp
8. (factor* adj2 associat*).mp
9. (factor* adj2 determin*).mp
10. (factor* adj2 predispos*).mp
11. (factor* adj2 precipitat*).mp
12. Exp epidemiology/
13. Epidemiolog*.mp
14. “Risk ratio*”.mp
15. “Odds ratio*”.mp
16. “Relative risk*”.mp
17. 1 or 2 or 3 or 4 or 5 or 6 or 7 or 8 or 9 or 10 or 11 or 12 or 13 or 14 or 15 or 16
18. exp multimorbidity/ or comorbidity/
19. (Multimorbid* or multi-morbid*).mp
20. (Comorbid* or co-morbid*).mp
21. “Multiple chronic disease*”.mp
22. “Multiple chronic condition*”.mp
23. “Multiple chronic ill*”.mp
24. “Multiple chronic disorder*”.mp
25. “Multiple chronic morbid*”.mp
26. “Multi* morbid*”.mp
27. (Multi* disease* or multi-disease*).mp
28. (Multi* condition* or multi-condition*).mp
29. (Multi* ill* or multi-ill*).mp
30. (Multi* disorder* or multi-disorder*).mp
31. “multi* health condition*”.mp
32. Multiple adj2 (disease* or condition* or ill* or morbid* or disorder*).mp
33. Concurrent adj2 (disease* or condition* or ill* or morbid* or disorder*).mp
34. ((Coexist* or co-exist*) adj2 (disease* or condition* or ill* or morbid* or disorder*).mp
35. ((Cooccur* or co-occur*) adj2 (disease* or condition* or ill* or morbid* or disorder*).mp
36. 18 or 19 or 20 or 21 or 22 or 23 or 24 or 25 or 26 or 27 or 28 or 29 or 30 or 31 or 32 or 33 or 34 or 35
37. exp India/
38. India*.mp
39. 37 or 38
40. 17 and 36 and 39

**EMBASE (OVID) (1974- August 03, 2022): 4730 records**

1. Exp risk factors/
2. (Risk factor* or risk-factor*).mp
3. Risk*.mp
4. Factor*.mp
5. (Risk assess* or risk-assess*).mp
6. Exp epidemiology/
7. Epidemilog*.mp
8. Exp risk assessment
9. “Risk quotient*”.mp
10. “Risk ratio*”.mp
11. “Odds ratio*”.mp
12. “Relative risk*”.mp
13. (factor* adj2 associate*).mp
14. (factor* adj2 determin*).mp
15. (factor* adj2 predispos*).mp
16. (factor* adj2 precipitat*).mp
17. 1 or 2 or 3 or 4 or 5 or 6 or 7 or 8 or 9 or 10 or 11 or 12 or 13 or 14 or 15 or 16
18. Exp multimorbidity/
19. (Multimorbid* or multi-morbid*).mp
20. (Comorbid* or co-morbid*).mp
21. Exp multiple chronic conditions/
22. “Multiple chronic disease*”.mp
23. “Multiple chronic condition*”.mp
24. “Multiple chronic disorder*”
25. “Multiple chronic ill*”
26. “Multiple chronic morbid*”
27. “Multiple chronic health condition*”
28. “Multiple chronic medical condition*”
29. ((Coexist* or co-exist*) adj2 (condition* or disease* or morbid* or disorder* or ill*).mp
30. (Concurrent adj2 (condition* or disease* or morbid* or disorder* or ill*)).mp
31. ((Cooccur* or co-occur*) adj2 (condition* or disease* or morbid* or disorder* or ill*).mp
32. (Simultaneous adj2 (condition* or disease* or morbid* or disorder* or ill*)).mp
33. 18 or 19 or 20 or 21 or 22 or 23 or 24 or 25 or 26 or 27 or 28 or 29 or 30 or 31 or 32
34. Exp India/
35. India*.mp
36. 34 or 35
37. 17 and 33 and 36

**PsycINFO (OVID) (1806- August 03, 2022): 258 records**

1. Exp risk factors/
2. Risk*.mp
3. (Risk factor* or risk-factor*).mp
4. Exp risk assessment/
5. (Risk assess* or risk-assess*).mp
6. “Risk ratio*”.mp
7. “odds ratio*”.mp
8. “Relative risk*”.mp
9. 1 or 2 or 3 or 4 or 5 or 6 or 7 or 8
10. Exp multimorbidity/ or comorbidity.mp
11. (multimorbid* or multi-morbid*).mp
12. (comorbid* or co-morbid*).mp
13. “Multiple chronic condition*”.mp
14. “Multiple chronic disease*”.mp
15. “Multiple chronic disorder*”.mp
16. “Multiple chronic ill*”.mp
17. “Multiple chronic morbid*”.mp
18. ((Coexist* or co-exist*) adj2 (condition* or disease* or morbid* or disorder* or ill*).mp
19. (Concurrent adj2 (condition* or disease* or morbid* or disorder* or ill*)).mp
20. ((Cooccur* or co-occur*) adj2 (condition* or disease* or morbid* or disorder* or ill*).mp
21. 10 or 11 or 12 or 13 or 14 or 15 or 16 or 17 or 18 or 19 or 20
22. Exp India.mp
23. India*.mp
24. 22 or 23
25. 9 and 21 and 24

**CINAHL Plus (EBSCOhost) (1945- August 03, 2022): 485 records**

S1 (MH “Risk Factors+”)

S2 “Risk*”

S3 “factor*”

S4 “risk factor* OR risk-factor*”

S5 (MH “Risk Assessment”)

S6 “Determinant*”

S7 (MH “Epidemiology+”)

S8 “factors N5 predisposing”

S9 “factors N5 associated”

S10 “factors N5 precipitating”

S11 (MH “Comorbidity/RF”)

S12 “multimorbidity”

S13 ““multiple chronic (condition* or disease* or ill* or morbid* or disorder*)””

S14 “multi-morbid* OR multi* morbid*” OR “comorbid* OR co-morbid*”

S15 ““coexist* OR co-exist* N5 (condition* or disease* or ill* or disorder* or morbid*)””

S16 ““concurrent N5 (condition* or disease* or ill* or disorder* or morbid*)””

S17 ““cooccur* OR co-occur* N5 (condition* or disease* or ill* or disorder* or morbid*)””

S18 (MH “India”)

S19 ((MH “India”)) AND (S1 OR S2 OR S3 OR S4 OR S5 OR S6 OR S7 OR S8 OR S9 OR S10)

S20 (((MH “India”)) AND (S1 OR S2 OR S3 OR S4 OR S5 OR S6 OR S7 OR S8 OR S9 OR S10)) AND (S13 OR S14 OR S15 OR S16 OR S17)

**ProQuest Dissertation and Theses (August 03, 2022): 112 records**

To make the search more comprehensive in ProQuest Dissertation and Theses database, for the search for the keyword “India” an extra filter was applied to limit the search to the abstract of the articles. The following search was run:

India AND (Multimorbidity OR multi-morbid* OR “multi* morbid*” OR “multiple chronic condition*” OR “multiple chronic disease*” OR “multiple chronic disorder*” OR “multiple chronic ill*” OR concurrent PRE/2 condition* OR concurrent PRE/2 disease* OR concurrent PRE/2 disorder* OR concurrent PRE/2 ill* OR concurrent PRE/2 morbid* OR (coexist* OR co-exist*) PRE/2 condition* OR (coexist* OR co-exist*) PRE/2 disease* OR (coexist* OR co-exist*) PRE/2 disorder* OR (coexist* OR co-exist*) PRE/2 ill* OR (coexist* OR co-exist*) PRE/2 morbid* OR (cooccur* or co-occur*) PRE/2 condition* OR (cooccur* or co-occur*) PRE/2 disease* OR (cooccur* or co-occur*) PRE/2 disorder* OR (cooccur* or co-occur*) PRE/2 ill OR (cooccur* or co-occur*) PRE/2 morbid*) AND (“Risk factor” OR risk* OR factor* OR risk-factor* OR factor* PRE/2 predispos* OR factor* PRE/2 precipitat* OR factor* PRE/2 associat* OR factor* PRE/2 determin* OR determinant* OR “risk ratio*” OR “odds ratio*” OR “relative risk*”)
