# Supplementary material for: Risk factors of multimorbidity among older adults in India: A systematic review and meta‐analysis
Source: Health Sci Rep. 2024 Feb 28;7(2):e1915. doi: 10.1002/hsr2.1915 (PMC10900089; doi:10.1002/hsr2.1915)
Supplement: Supplementary file 2 — Supporting information. [file HSR2-7-e1915-s001.docx]

**Supplementary Material S2: Reasons for exclusion of full-text studies**

**Ineligible population (n=04)**

1. Hossain B, Govil D, Sk MIK. Persistence of multimorbidity among women aged 15-49 years in India: an analysis of prevalence, patterns and correlation. *International Journal of Public Health*. 2021;66: 601591.
2. Low LL, Kwan YH, Ko MSM, Yeam CT, Lee VSY, Tan WB, Thumboo J. Epidemiologic characteristics of multimorbidity and sociodemographic factors associated with multimorbidity in rapidly ageing Asian country. *JAMA Network Open*. 2019;2(11): e1915245.
3. Puri P, Kothavale A, Singh SK, Pati S. Burden and determinants of multimorbidity among women in reproductive age group: a cross-sectional study based in India. *Wellcome Open Research*. 2020;5: 275.
4. Mishra VK, Srivastava S, Muhammad T, Murthy PV. Population attributable risk for multimorbidity among adult women in India: do smoking tobacco, chewing tobacco and consuming alcohol make a difference. *PLoS One*. 2021;16(11): e0259578.

**Ineligible outcomes (n=17)**

1. Arokiasamy P, Uttamacharya, Kowal P, Capistrant BD, Gildner TE, et al. Chronic noncommunicable disease in 6 low-and middle-income countries: findings from wave 1 of the World Health Organization’s Study on Global Ageing and Adult Health (SAGE). *American Journal of Epidemiology.* 2017;185(6): 414-428.
2. Barik BS, Dash ST, Barik M, Yadav VS, Hussain T, Pati S. Study of the morbidity pattern among geriatric patients attending a secondary care hospital in Bhubaneswar, Odisha. *Ageing Medicine and Healthcare*. 2022;13: 78-86.
3. Brinda E, Attermann J, Gerdtham UG, Enemark U. Socio-economic inequalities in health and health service use among older adults in India: results: results from the WHO Study on Global AGEing and adult health survey. *Public Health*. 2016;141: 32-41.
4. Kshatri JS, Palo SK, Bhoi T, Barik SR, Pati S. Association of multimorbidity on frailty and dependence among an elderly rural population: findings from the AHSETS study. *Mechanisms of Ageing and Development*. 2020;192: 111384.
5. Pati Sandipana, Schellevis FG. Prevalence and pattern of comorbidity among type 2 diabetic attending urban primary healthcare centres at Bhubaneswar (India). *PLoS One*. 2017;12(8): e0181661.
6. Raman VK, Suresh KP. Prevalence of hypertension and diabetes morbidity among adults in a few urban slums of Bangalore city, determinants of its risk factors and opportunities for control- a cross-sectional study*. Journal of Family Medicine and Primary Care.* 2020;9(7): 3264-3271.
7. Sharma D, Mazta SR, Parashar A. Morbidity pattern and health-seeking behaviour of aged population residing in Shimla hills of North India: a cross-sectional study. *Journal of Family Medicine and Primary Care*. 2013;2(2): 188-193.
8. Adaji EE, Ahankari AS, Myles PR. An investigation to identify potential risk factors associated with common chronic diseases among older population in India. *Indian Journal of Community Medicine.* 2017;42(1): 46-52.
9. Joshi K, Kumar R, Avasthi A. Morbidity profile and its relationship with disability and psychological distress among elderly people in Northern India*. International Journal of Epidemiology*. 2003;32(6): 978-987.
10. Bhatt R, Gadhvi MS, Sonaliya KN, Solanki A, Nayak H. An epidemiological study of morbidity pattern among the elderly population in Ahmedabad, Gujrat. *National Journal of Community Medicine*. 2011;2(2).
11. Vadrevu L, Kumar V, Kanjilal B. Rising challenge of multiple morbidities among the rural poor in India: a case of the Sundarbans in West Bengal. *International Journal of Medical Science and Public Health*. 2016;5(2): 343.
12. Sahukaiah S, Shenoy A, Vijayakumar BC. An epidemiological study of prevalence of morbidity patterns among geriatric age group in an urban slum of Mumbai. *International Journal of Medical Science and Public Health.* 2015;4(7): 883.
13. Kalathingal HK, Xavier AU. A study on morbidity profile among elderly persons in rural area of Kozhikode district. *International Journal of Medical Science and Public Health*. 2019;9(4): 137-140.
14. Eram U, Nawab T, Khalique N, Akhouri DD. Study of morbidity pattern in geriatric population in rural areas of Aligarh. *International Journal of Current Trends in Engineering and Technology*. 2016;2(6).
15. Kakkar R, Aggarwal P, Kandpal SD, Bansal SK. An epidemiological study to assess morbidity profile among geriatric population in district Dehradun*. Indian Journal of Community Health*. 2013;25(1): 39-44.
16. Mohapatra A, Handoo SK, Gambhir IS, Mohapatra SC. A study of non-communicable morbidity pattern in geriatric patients attending a referral railway hospital in Allahabad, Uttar Pradesh. *National Journal of Community Medicine*. 2011;2(2).
17. Jadhav VS, Mundada VD, Gaikwad AV, Doibale MK, Kulkani AP. A study of morbidity profile of geriatric population in the field practice area of rural health training centre, paithan of government medical college, Aurangabad. *IOSR Journal of Pharmacy*. 2012;2(2): 184-188.

**Ineligible study designs (n=02)**

1. Morgan SA. Multimorbidity: its prevalence and impact in middle-income countries. A multicounty comparison using household surveys and qualitative methods [thesis on internet]. University of Southampton; 2017. [Cited September 07, 2022].
2. Singh K, Patel SA, Biswas S, Shivashankar R, Kondal D, Ajay VS, Anjana RM, Fatmi Z, et al. Multimorbidity in South Asian adults: prevalence, risk factors and mortality. *Journal of Public Health*. 2019;41(1): 80-89.

**Ineligible population + ineligible outcomes (n=08)**

1. Geldsetzer P, Neve DJW, Mohan V, Prabhakaran D, Roy A, Tandon N, Davies JI, Vollmer S, Barighausen T, Prenissl J. Health system performance for multimorbidity cardiometabolic disease in India: a population-based cross-sectional study. *Global heart.* 2022;17(1): 07.
2. Bansode B, Prasad JB. Burden of comorbidities among diabetic patients in Latur, India. *Clinical Epidemiology and Global Health*. 2022;13: 100957.
3. Agarwal AK, Gupta G, Marskole P, Agarwal A. A study of patients suffering from tuberculosis and tuberculosis-diabetes comorbidity in Revised National Tuberculosis Control Program Centers of Northern Madhya Pradesh, India*. Indian Journal of Endocrinology and Metabolism.* 2017;21(4): 507-576.
4. Mini GK, Mohan M, Sarma PS, Thankappan KR. Multi-morbidity and blood pressure control: results of a cross-sectional study among school teachers in Kerala, India. *Indian Journal of Public Health.* 2021;65(2): 190-193.
5. Patel V, Jaisoorya TS, Kamble N, Yadav R, Thennarassu K, Pal PK, Reddy JYC. Prevalence and correlates of psychiatric comorbidity and multimorbidity in Parkinson’s disease and atypical Parkinsonian syndrome. *Journal of Geriatric Psychiatry and Neurology*. 2017.
6. Pati S, Bhattacharya S, Swain S. Prevalence, and patterns of multimorbidity among human deficiency virus positive people in Odisha, India: an exploratory study. *Journal of Clinical Diagnostic and Research*. 2017;11(6): LC10-LC13.
7. Stubbs B, Vancampfort D, Veronese N, Schofield P, Lin PY, Tseng PT, Solmi M, Thompson T, Carvalho AF, Koyanagi Ai. Multimorbidity and perceived stress: a population-based cross-sectional study among older adults across six low-and middle-income countries. *Maturitas*. 2018;107: 84-91.
8. Joshi R, Santoshi JA, Rai N, Pakhare A. Prevalence, and patterns of coexistence of multiple chronic conditions: a study from Indian urban outpatient setting. *Journal of Family Medicine and Primary Care*. 2015;4(3): 411-415.

**Relevant data could not be extracted (n= 16)**

1. Gupta P, Patel SA, Sharma H, Jarhyan P, Sharma R, Prabhakaran D, Tandon N, Mohan S. Burden, patterns, an impact of multimorbidity in North India: findings from a rural population-based study. *BMC Public Health*. 2022;22(1): 1101.
2. Agrawal G, Patel SK, Agarwal AK. Lifestyle health risk factors and multiple non-communicable diseases among the adult population in India: a cross-sectional study. *Journal of Public Health.* 2016;24(4): 317-324.
3. Rohini C, Jeemon P. Prevalence, and patterns of multi-morbidity in the productive age group of 30-69 years: a cross-sectional study in Pathanamthitta District, Kerala. *Wellcome Open Research*. 2020;5: 233.
4. Khan MR, Malik MA, Akhtar SN, Yadav S, Patel R. Multimorbidity and its associated risk factors among the older adults in India. *BMC Public Health*. 2022;22(1): 746.
5. Romano E, Ma R, Vancampfort D, Firth J, Nobrega MF, Haro JM, Stubbs B, Koyanagi Ai. Multimorbidity and obesity in older adults from six low-and middle-income countries*. Preventive Medicine*. 2021;153: 106816.
6. Garin N, Koyanagi A, Chatterji S, Tyrovolas S, Olaya B, Leonardi M, Lara E, Koskinen S, Adamczyk BT, Mateos JLA, Haro JM. Global multimorbidity patterns: a cross-sectional, population-based, multi-country study*. Journals of Gerontology Series A- Biological Sciences & Medical Sciences*. 2016;71(2): 205-214.
7. Pati S, Swain S, Hussain MK, Kadam S, Salisbury C. Prevalence, correlates, and outcomes of multimorbidity among patient attending primary care in Odisha, India. *Annals of Family Medicine*. 2015;13(5): 446-450.
8. Pati S, Swain S, Metsemakers J, Knottnerus JA, Akker MVD. Pattern and severity of multimorbidity among patients attending primary care setting in Odisha, India. *PLoS One*. 2017;12(9): e0183966.
9. Pati S, Swain S, Knottnerus JA, Metsemakers J, Akker MVD. Health related quality of life in multimorbidity: a primary-care based study from Odisha, India. *Health Quality Life Outcomes.* 2019;16: 116.
10. Pati S, Swain S, Knottnerus JA, Metsemakers J, Akker MVD. Magnitude and determinants of multimorbidity and health care utilization among patients attending public versus private primary care: a cross-sectional study from Odisha, India. *International Journal for Equity in Health*. 2020;15: 57.
11. Pati S, Mahapatra P, Dwivedi R, Athe R, Sahoo KC, Samal M, Das RC, Hussain MK. Multimorbidity and its outcomes among patients attending psychiatric care settings: an observational study from Odisha, India. *Front Public Health*. 2020;8: 616480.
12. Pati S, Mahapatra P, Kanungo S, Uddin A, Sahoo KC. Managing multimorbidity (multiple chronic diseases) amid covid-19 pandemic: a community-based study from Odisha, India. *Front Public Health*. 2020;8: 584408.
13. Palo SK, Swain S, Priyadarshini S, Behera B, Pati S. Epidemiology of obesity and its related morbidities among rural population attending a primary health centre of Odisha, India. *Journal of Family Medicine and Primary Care*. 2019;8(1): 203-208.
14. Reddy MM, Zaman K, Yadav R, Yadav P, Kumar K, Kant R. Prevalence, associated factors, and health expenditure of noncommunicable disease multimorbidity- findings from Gorakhpur health and demographic surveillance system. *Front Public Health*. 2022;10: 842561.
15. Soji DJ, Lordson J, Mini GK. Multimorbidity patterns among rural adults with type-2 diabetes mellitus: a cross-sectional study in Kerala, India. *WHO Southeast Asia Journal of Public Health*. 202;10(1): 32-36.
16. Pati S, Sinha R, Panda M, Puri P, Pati Sandipana. Profile of multimorbidity in outpatients attending public healthcare settings: a descriptive cross-sectional study from Odisha, India. *Journal of Family Medicine and Primary Care*. 2021;10(8): 2900-2914.

**No additional data could be extracted (n= 14)**

1. Puri P, Singh SK, Pati S. Temporal dynamics, patterns and correlates of single and multimorbidity in India, 1994-2018. *Journal of comorbidity*. 2021;11: 26335565211062756.
2. Boro B, Saikia N. Association of multimorbidity and physical activity among older adults in India: an analysis from the Longitudinal Ageing Survey of India (2017-2018). *BMJ Open*. 2022;12(5): e053989.
3. Chauhan S, Srivastava S, Kumar P, Patel R. Decomposing urban-rural differences in multimorbidity among older adults in India: a study based on LASI data*. BMC Public Health*. 2022;22(1): 502.
4. Mini GK, Thankappan KR. Pattern, correlates and implications of non-communicable disease multimorbidity among older adults in selected Indian states: a cross-sectional study. *BMJ Open*. 2017;7(3): e013529.
5. Sinha A, Kerketta S, Ghosal S, Kanungo S, Pati S. Multimorbidity among urban poor in India: findings from LASI, wave 1. *Frontiers in Public Health*. 2022;10: 881967.
6. Srivastava S, Joseph VKJ, Drishti D, Muhammad T. Interaction of physical activity on association of obesity-related measures with multimorbidity among older adults: a population-based cross-sectional study in India. *BMJ Open*. 2021;11(5): e050245.
7. Himanshu, Talukdar B. Prevalence of multimorbidity (chronic non communicable diseases) and associated determinants among elderly in India. *Demography India*. 2017; special issue:69-76.
8. Arokiasamy P, Uttamacharya, Jain K, Biritwum RB, Yawson AE, Wu F, Guo Y, et al. The impact of multimorbidity on adult physical and mental health in low-and middle-income countries: what does the study on global ageing and adult health (SAGE) reveal. *Medicine for Global Health*. 2015;13: 178.
9. Puri P, Pati S. Exploring the linkage between non-communicable disease multimorbidity, health care utilization and expenditure among aboriginal older adult population in India. *International Journal of Public Health*. 2022;67: 1604333.
10. Puri P, Singh SK, Pati S. Identifying non-communicable disease multimorbidity patterns and associated factors: a latent class analysis approach. *BMJ Open*. 2022;12(7): e053981.
11. Puri P, Sinha A, Mahapatra P, Pati S. Multimorbidity among midlife women in India: well-being beyond reproductive age. *BMC Women’s Health*. 2022;22: 117.
12. Smith L, Shin JI, Butler L, Barnett Y, Oh H, Jacob L, Kostev K, Veronese N, Soysal P, Tully M, Sanchez GFL, Koyanagi Ai. Physical multimorbidity and depression: a medication analysis of influential factors among 34,129 aged ≥50 years from low-and middle-income countries. *Depression and Anxiety*. 2022;39(5): 376-386.
13. Puri P, Singh SK. Patterns and predictors of non-communicable disease multimorbidity among older adults in India: evidence from longitudinal ageing study in India (LASI), 2017-2018. *Journal of Public Health Policy*. 2022;43(1): 109-128.
14. Sinha A, Kerketta S, Ghosal S, Kanungo S, Lee JT, Pati S. Multimorbidity and complex multimorbidity in India: findings from the 2017-2018 Longitudinal Ageing Study in India (LASI). *International Journal of Environment Research & Public Health*. 2022;19(15): 9091.
